# Is it possible to implement a rare disease case-finding tool in primary care? A UK-based pilot study

**DOI:** 10.1186/s13023-022-02216-w

**Published:** 2022-02-16

**Authors:** Orlando Buendia, Sneha Shankar, Hadley Mahon, Connor Toal, Lara Menzies, Pradeep Ravichandran, Jane Roper, Jag Takhar, Rudy Benfredj, Will Evans

**Affiliations:** Mendelian, 239 Old St, London, EC1V9EY UK

**Keywords:** Rare disease, Primary care, Electronic health records, Database analysis

## Abstract

**Introduction:**

This study implemented MendelScan, a primary care rare disease case-finding tool, into a UK National Health Service population. Rare disease diagnosis is challenging due to disease complexity and low physician awareness. The 2021 UK Rare Diseases Framework highlights as a key priority the need for faster diagnosis to improve clinical outcomes.

**Methods and results:**

A UK primary care locality with 68,705 patients was examined. MendelScan encodes diagnostic/screening criteria for multiple rare diseases, mapping clinical terms to appropriate SNOMED CT codes (UK primary care standardised clinical terminology) to create digital algorithms. These algorithms were applied to a pseudo-anonymised structured data extract of the electronic health records (EHR) in this locality to "flag" at-risk patients who may require further evaluation. All flagged patients then underwent internal clinical review (a doctor reviewing each EHR flagged by the algorithm, removing all cases with a clear diagnosis/diagnoses that explains the clinical features that led to the patient being flagged); for those that passed this review, a report was returned to their GP. 55 of 76 disease criteria flagged at least one patient. 227 (0.33%) of the total 68,705 of EHR were flagged; 18 EHR were already diagnosed with the disease (the highlighted EHR had a diagnostic code for the same RD it was screened for, e.g. Behcet’s disease algorithm identifying an EHR with a SNOMED CT code Behcet's disease). 75/227 (33%) EHR passed our internal review. Thirty-six reports were returned to the GP. Feedback was available for 28/36 of the reports sent. GP categorised nine reports as "Reasonable possible diagnosis" (advance for investigation), six reports as "diagnosis has already been excluded", ten reports as "patient has a clear alternative aetiology", and three reports as "Other" (patient left study locality, unable to re-identify accurately). All the 9 cases considered as "reasonable possible diagnosis" had further evaluation.

**Conclusions:**

This pilot demonstrates that implementing such a tool is feasible at a population level. The case-finding tool identified credible cases which were subsequently referred for further investigation. Future work includes performance-based validation studies of diagnostic algorithms and the scalability of the tool.

## Background

Rare diseases (RD) are individually rare but collectively common [1], with an estimated 6000–8000 RD they affect 3.5–5.9% of the population or 263–446 million persons globally [2]. RD are heterogeneous in aetiology, frequently chronic and debilitating [3]. There is no universal definition of a rare disease, with most legislative frameworks using point prevalence. In the UK and the European Union (EU), a rare disorder is defined as affecting fewer than 1 in 2000 persons [4, 5]. 71.9% are considered genetic diseases, and 69.9% have a paediatric-onset [2]. Of all RD, 149 diseases (4.2%) have a prevalence in the range of 1–5 per 10,000, these account for 77.3–80.7% of the total population of patients affected. Collectively RD are a significant burden to healthcare systems and society, in the US the annual economic burden of 379 RD, with a combined incidence of 15.5 million, was estimated to be $966 billion in 2019 [6].

Rare disease diagnosis is challenging, patients frequently remain without a correct diagnosis for extended periods. This hunt for a diagnosis has its own term: the diagnostic odyssey [7]. During this diagnostic odyssey, patients typically experience numerous primary care visits, specialist clinic reviews, investigations, interventions, misdiagnoses and inappropriate treatments [8]. A cohort of patients with RD in the UK and US reported a diagnostic delay of an average of 5.6 and 7.6 years respectively, with patients typically visiting eight physicians (four primary care and four specialists) and receiving two to three misdiagnoses [9]. Similarly, an EU survey reported that 40% of patients with RD were initially incorrectly diagnosed, and a quarter experienced a diagnostic odyssey of more than 5 years [10].

The reason for this diagnostic delay is multifactorial. No individual clinician can be expected to know all RD, and the adage "When you hear hoofbeats, do not expect to see Zebras" [11], describes a well-held approach to considering the differential diagnosis of a clinical problem, but is not helpful for diagnosing patients with RD. Enabling clinicians, especially those in primary care, to identify unusual patterns and revisit diagnoses is crucial to reducing the diagnostic odyssey for patients with RD [12].

Early diagnosis is central to achieving better patient outcomes [13]. It enables an improved assessment of prognosis, optimization of care, access to therapies, linkage to patient organisations, easier access to social and educational support, as well as more accurate disease information [12]. It also brings clarity and understanding to the challenging, puzzling and costly diagnostic odyssey for patients and their families [14]. Furthermore, an accurate diagnosis enables the patient to contribute to the broader understanding of their disease, through for example patient registries, engagement with research and therapy development [15].

In the UK, addressing this diagnostic odyssey is the first of four key priorities in the UK Rare Diseases Framework, published in January 2021. The priority ‘Helping rare disease patients get a final diagnosis faster’ is underpinned by five themes and proposes using data and digital technologies as a solution to enable a more timely diagnosis [16].

## Methods

**Fig. 1:**
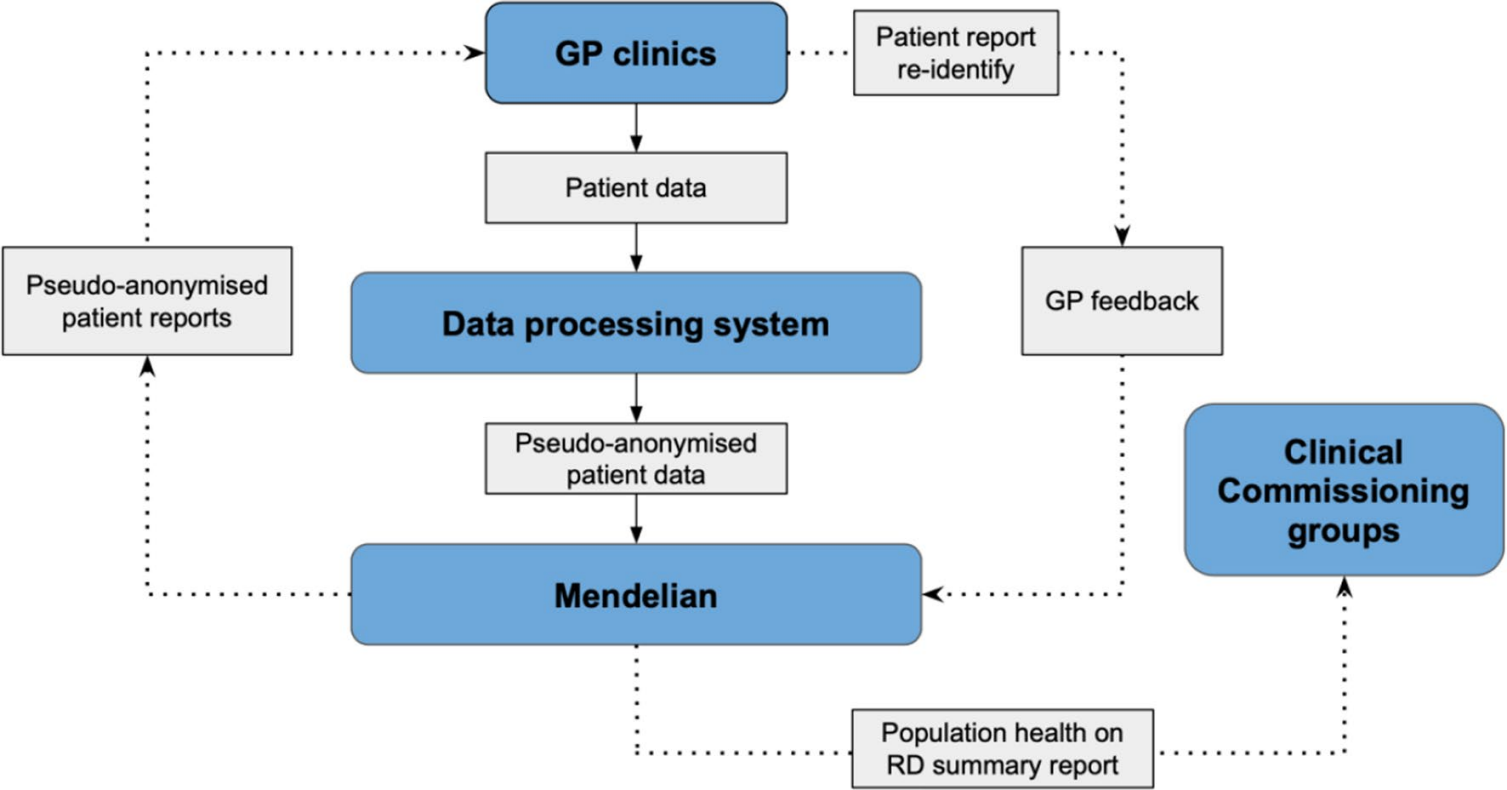
MendelScan data flow and systems integration

Mendelian is a UK-based health data analytics company focused on shortening the diagnostic odyssey of rare and hard-to-diagnose diseases. Mendelian has developed a digital case-finding tool, “MendelScan”, that can analyse structured clinical vocabulary, such as SNOMED CT codes [17] from primary care electronic health records (EHR) and highlight patterns of data that correspond to an increased likelihood of the patient being affected by certain RD. This enables the identification of those at risk and assists their clinician in accessing the correct diagnostic pathway. The MendelScan system is summarised in Fig. 1.

The pilot study took place between January 2019 and October 2020. The primary objective was to assess the feasibility of applying MendelScan with seventy-six rare disease algorithms (see “Appendix 1”), in a primary care environment in the lower lea valley (LLV) primary care GP Federation.

**Fig. 2:**
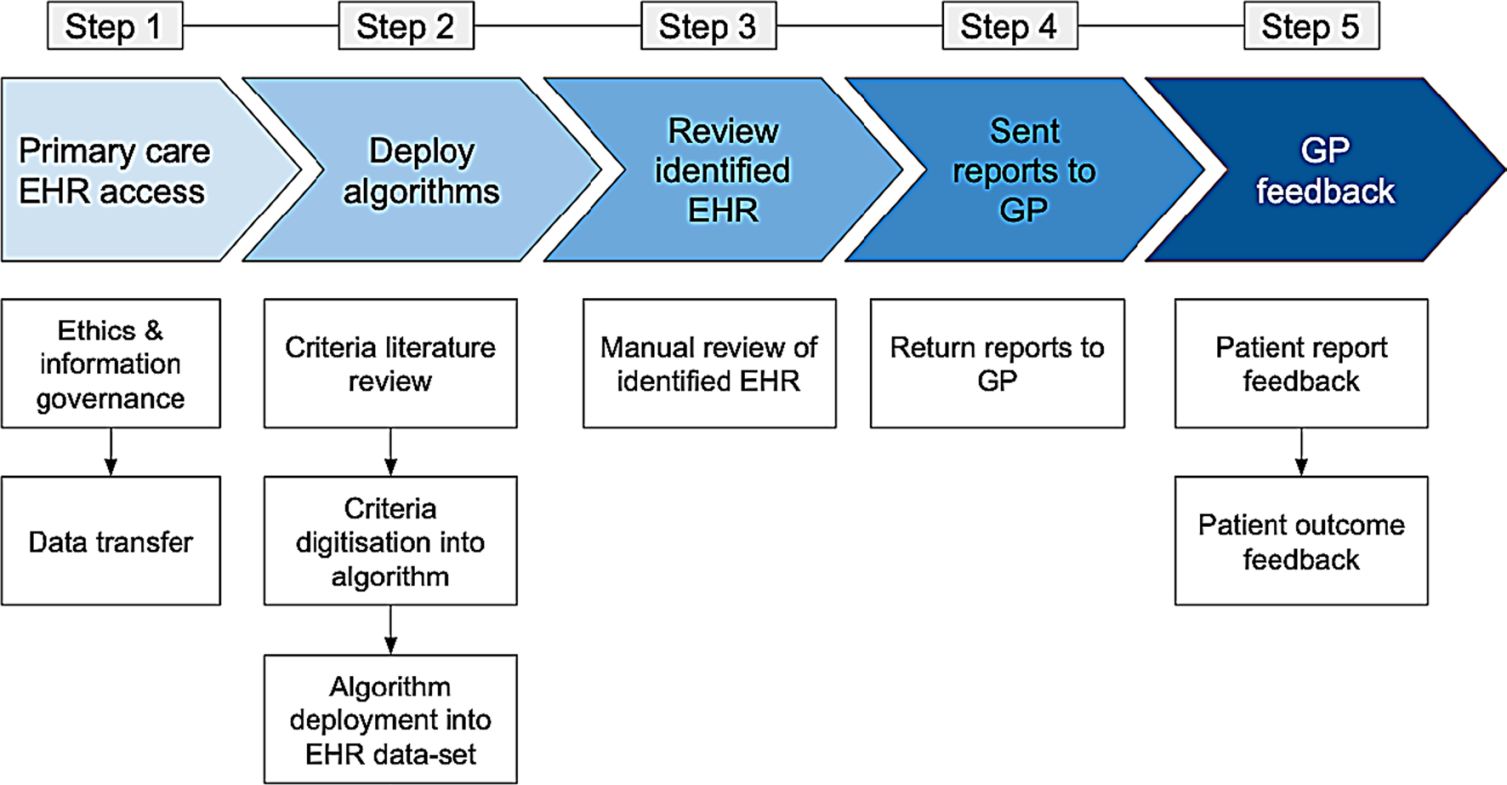
Overview RD case-finding digital tool “MendelScan” implementation steps (Methods)

The process for delivering MendelScan into the selected primary care federation involved establishing agreements, deploying the algorithms into a pseudonymised data set, manually reviewing the EHR identified by the algorithm, delivering the reports to GP and collecting their feedback. Figure 2 summarises the implementation process.

### Primary care EHR access

#### Ethics and information governance

To facilitate data access and establish confidence in this study, an independent ethical analysis of this approach was commissioned [18]. Building on the outcome and recommendations of this report, and in compliance with information governance legislation, a data-sharing agreement (DSA) was agreed between stakeholders (Mendelian, Medeanalytics, East and North Hertfordshire NHS trust and Lea valley primary care network). The DSA is a contract that stipulates the rules regarding the usage and handling of data. Finally, a data protection impact assessment (DPIA) was drafted, identifying and minimising the data protection risks of the project [19].

#### Data transfer

Data transfer involved Medanalytics creating a data set of patients’ EHR, removing personal identifiers, and individuals who had opted out of sharing through the national data opt-out. For the remainder EHR a pseudonym, with a unique numeric identifier was created. This pseudonymised dataset of records was sent to Mendelian for analysis.

### Algorithm deployment

Not all of the 7000–9000 rare diseases are appropriate for MendelScan. Mendelian developed a stepped approach to stratify which rare diseases are more likely to be suitable for primary care records analysis:

1. Analysing the suitability of the RD by scoring the features of the disease, the benefit of early diagnosis and the likelihood that the relevant clinical characteristics would be captured in the primary care EHR.
2. For a disease to be deemed suitable, it had to meet the three compulsory core variables, with diseases then prioritised on the response to the three optional variables. (See Table 1).
3. Performing a literature review, searching for peer-reviewed screening or diagnostic criteria for the selected RD.
4. For a criteria to be deemed suitable, it had to meet the two compulsory core variables, with the diseases then prioritised on the response to the three optional variables. (See Table 2).
5. Digitising the selected criteria into a numeric algorithm using structured data codes (SNOMED CT), across a range of EHR code types. (see Table 3) We did not interrogate data held in unstructured formats (free text) such as letters or consultation notes.

**Table 1:** Variables for RD suitability of primary care records analysis

Disease scoring	
Core variables	Metric
Is there an absence of significant mortality (> 30%) before five years of age?	Yes/no
Does the disease have signs and symptoms that are progressive and potentially missed?	Yes/no
Three or more clinical features likely to be encoded in the primary care EHR?	Yes/no
**Optional variable**	**Metric**
Does the disease have a high-specialised service pathway in the NHS?	Yes/no
Is the disease multisystemic? (≥ than three organ systems involved)	Yes/no
Point prevalence greater than 1:100,000?	Yes/no

**Table 2:** Variables for criteria suitability of primary care records analysis

Criteria scoring	
Core variables	Metric
Has a criterion been identified in the literature review?	Yes/no
Does the criterion have findings that will be captured in structured data in the primary care EHR?	Yes/no

Optional variable	Metric
Does the criterion use a classification/scoring system?	Yes/no
Do the specific features/findings at the criterion are explained in the peer-reviewed article?	Yes/no
Are there further studies that validate the performance of the criterion?	Yes/no

**Table 3:** Electronic health record (EHR) code types used

EHR code type	Description
Vitals	Physiological values such as blood pressure, weight, height, and BMI
Demographics	Patients demographic information such as age, sex and ethnicity
Problem list	Patients list of active medical issues
Diagnosis	Diagnosis and diagnostic codes
Referrals	Referral ordered and admissions
Medications	Medications ordered and currently taken
Lab results	Numerical results of any laboratory tests

The MendelScan case-finding tool checked the seventy-six disease algorithms against the pseudonymised EHR data extracts flagging patients who met the algorithms’ threshold of being at risk of the disease. Flagged patients’ structured EHR were then reviewed by a clinician, and a report was returned to the GP if a plausible alternative explanation for the clinical features could not be found.

### Internal review of identified cases’ EHR

We performed an anonymous, two‐round manual review process for each EHR identified by any of the seventy-six algorithms deployed. In round one, a medical doctor reviewed each EHR and assigned to each case one of three outcomes:

- *Rule-in* The medical doctor considers that there is enough clinical evidence to suspect the highlighted RD for this case.
- *Rule-out* The medical doctor considers there are other diagnoses recorded in the EHR that explains the highlighted features.
- *Already diagnosed* The highlighted EHR has a diagnostic code for the same RD it was screened for.

**Fig. 3:**
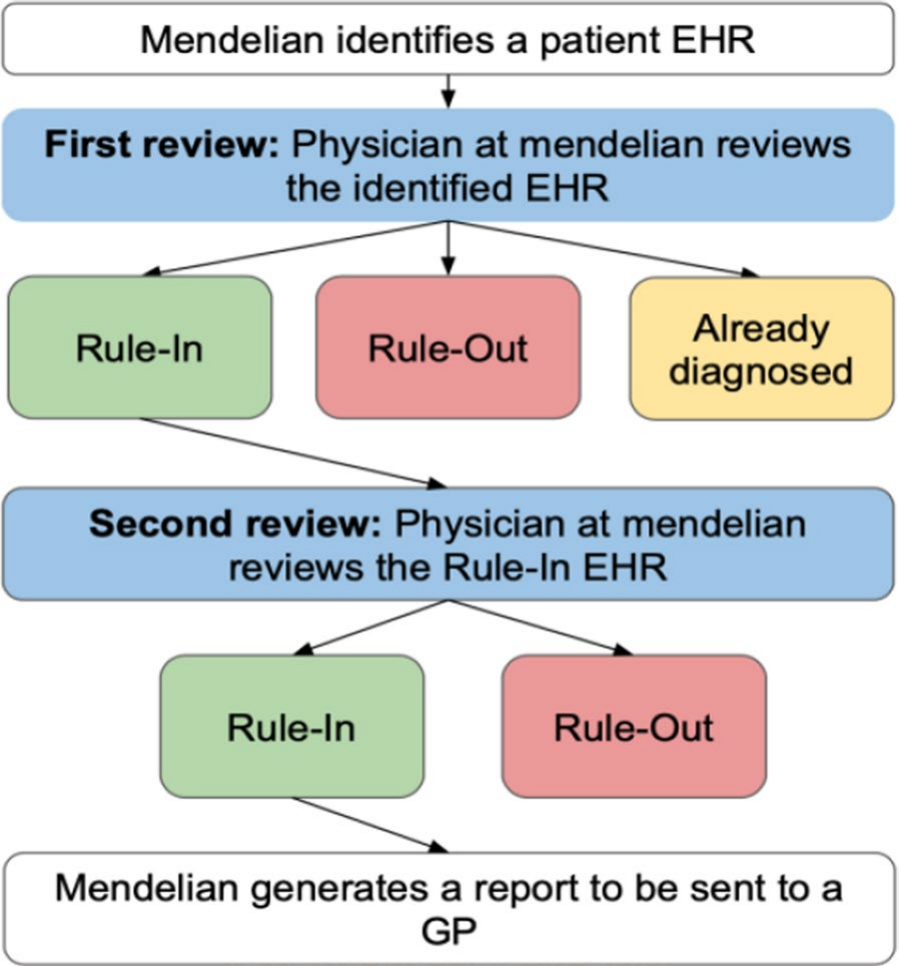
Internal review process methods

In round two, rule-in cases were further reviewed by a GP, geneticist or an expert in a particular rare disease and further assigned a rule-in or rule-out outcome. For each rule-in case, a patient report was generated and sent to their GP practice. The review process is summarised in Fig. 3.

### Returning reports to GP

A report for each of the ‘rule-in’ patients was returned to their GP by email. The report included the unique patient identifier, to enable re-identification and matching to the patient’s full EHR, an explanation of the condition, the reasons why this patient was flagged, and suggested next steps.

### GP feedback on reports

Feedback from the GP was requested at two stages. The first, ‘patient report feedback’, was requested at the time the GP completed evaluating the patient’s report and EHR. This consisted of an online questionnaire accessed through a link on each patient report (“Appendix 2”).

The first question asked the GP the main outcome of the report. See Table 4.

**Table 4:** Patient report feedback

Please indicate which options below best describe this report. Tick box multiple answer questions	
Reasonable possible diagnosis (Advance for further evaluation)	
Diagnosis has already been excluded (Disease highlighted in the report was already studied)	
Patient has a clear alternative diagnosis that explains the clinical features flagged	
Patient has left GP practice	
Unable to accurately identify patient	

**Fig. 4:**
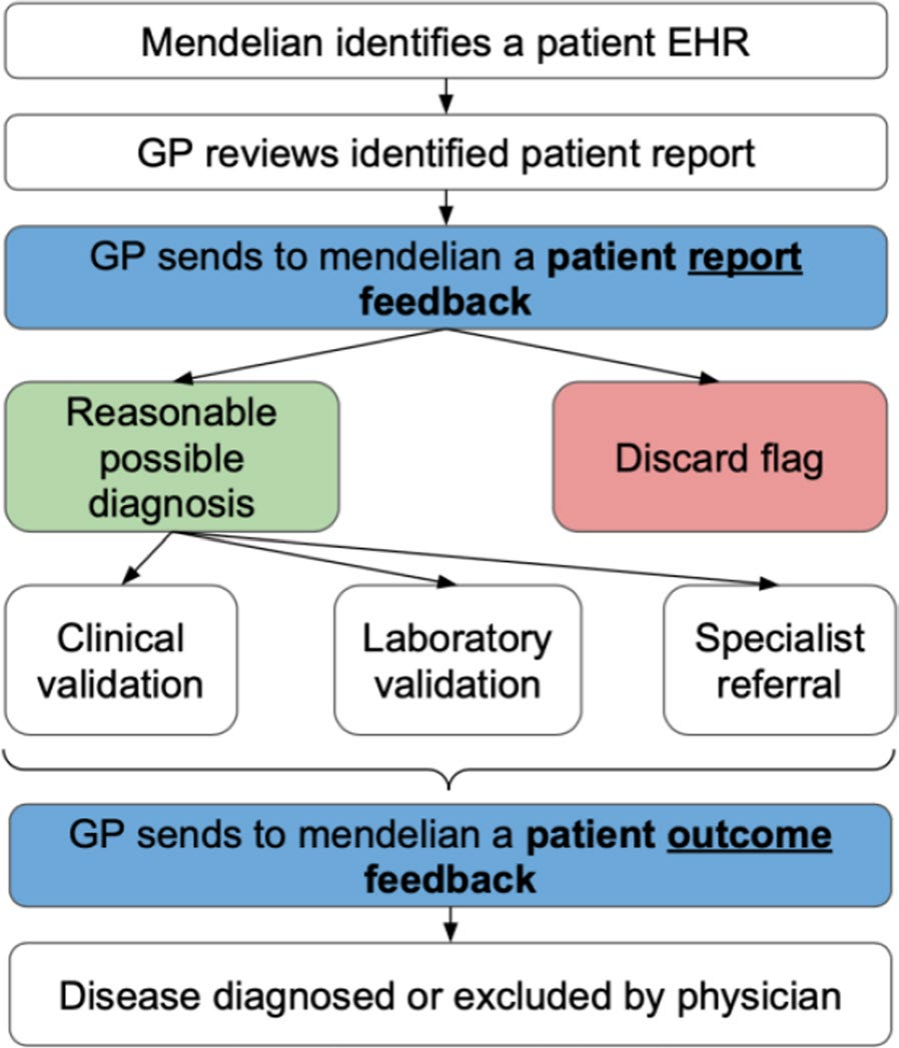
Feedback flowchart

The second, patient outcome feedback was requested 3 months later requesting the result of those advanced for further evaluation Fig. 4.

## Results

**Fig. 5:**
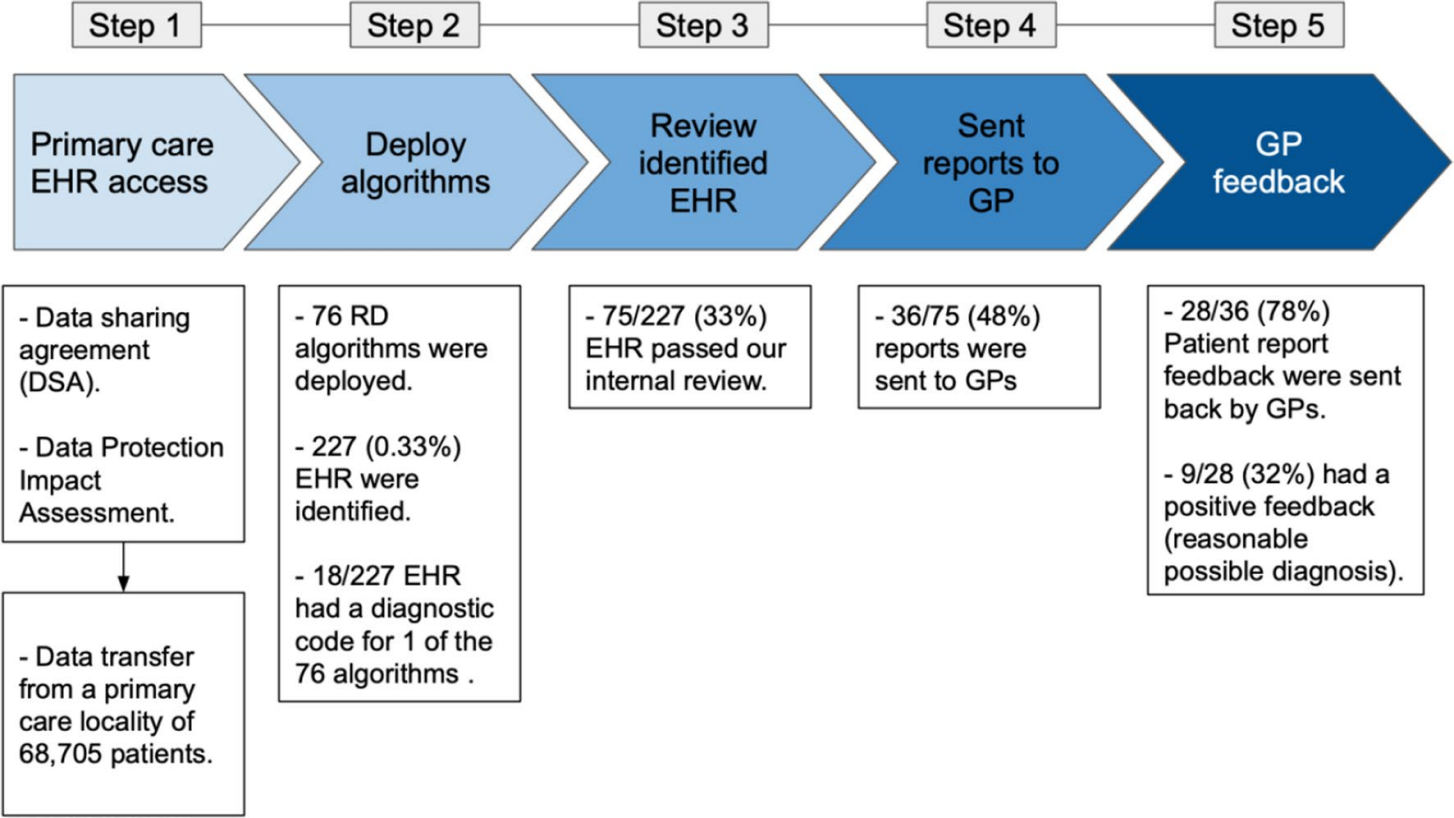
Results overview of RD “MendelScan” implementation steps

Delivering MendelScan into a primary care locality involved a process starting with setting up the agreements through to receiving feedback from the reports sent to GPs. The main results are summarised in Fig. 5.

### Primary care EHR access

#### Ethics and information governance

The ethics report helped to clarify challenges and potential risks in data management for stakeholders to minimise. The information governance process (Data Sharing Agreement and the Data Protection Impact Assessment) took almost a year to complete. There were no standard documents to enable data transfer for commercial service providers in the NHS. With no standardised documents nor any previous experience of such processes in either Mendelian nor the LLV, this took longer than anticipated to agree on the legal and information governance documentation.

#### Data transfer

The process of data transfer led to about a one-third reduction in the number of EHR. This was either due to no clinical events being reced in a patient's EHR, the death of the patient, deregistration or issues related to the quality of the data (incomplete or an unsound record). After the data cleaning process, the de-anonymised data set included 68,705 patients’ EHR.

### Algorithm deployment

We analysed 259 RD. 76 RD passed the stepped approach for algorithm development and adoption. All 76 algorithms were deployed successfully into the de-anonymised data set. 55 of the 76 algorithms identified at least one EHR. 227 EHR (0.33% of the total population) were identified in total. 18 of the 227 EHR had an existing diagnostic code for the flagged RD. In the total population, there were 152 patients’ EHR with a diagnostic code for one of the 76 rare diseases.

### Internal review of identified EHR

75 of the 227 EHR (33%) passed our internal review process.

### Send reports to GP

36 of the 75 (48%) patient reports were returned to the GP practices. The return of reports was batched to manage GP workload (a maximum of 5 reports per week per GP practice). A GP Federation Research Nurse, with access to the full EHR across multiple practices, did an initial review of reports for all but one of the practices in the study. The reviewing for this single practice was done by a GP at the practice.

39 (52%) of the reports were not delivered. This was due to clinical pressures associated with the COVID-19 pandemic, and restructuring of the primary care organisation leading to the need to review contracting and data sharing agreements. Feedback regarding the process of returning reports included that: the batching of reports into fives was welcomed, enabling the work of reviewing cases to be planned efficiently; the reports were re-identified within the primary care data system successfully; the reports were found to be clearly laid out with the reasons for flagging and supporting evidence easy to interpret; the layout enabled a quick and targeted approach to challenge/confirm the conclusions.

### GP feedback on reports

The initial feedback, “Patient report feedback”, was available for 28 of the 36 reports sent to the GP. The 8 outstanding patients were lost to follow up due to delays, the result of clinical pressures and organisational restructuring (Table 5).

**Table 5:** Patient report feedback results

	Reasonable possible diagnosis (advanced for investigation)	Diagnosis has already been excluded	GP believes the patient has a clear alternative aetiology	Other (Patient no longer at the practice or unable to correctly identify patient record)
Number of EHR	9	6	10	3

GPs re-identified 27/28 (96%) of the reports within the primary care data system, the missing patient’s record could not be reidentified-this was presumed to be because the patient had left the practice. The time taken to review a report in primary care was between ten and thirty minutes. This time was dependent upon the disease itself, the patient complexity and the clinician’s familiarity with both the patient and disease. The follow-up feedback (patient outcome feedback) was available for six of the nine patients. The three patients, for whom data was not available, were lost to follow up due to primary care restructuring with their practices moving from the organisation contracted to perform the pilot study during the project. Time for feedback varied from one to eight months depending on the engagement of the primary care practice and the nature of the confirmatory test performed. For example, alpha-1-antitrypsin was quickly excluded with a blood test in primary care, whereas 22q11 deletion syndrome required a referral to clinical genetics, a referral that was further delayed due to the pandemic. Summary outcomes are detailed in Table 6.

**Table 6:** Patient outcome feedback results

Patient EHR	Suggested disease	Action taken by GP	Outcome
1	Classic Homocystinuria	Discuss with patient	Not available
2	Fabry Disease	Discuss with patient	Not available
3	Alpha-1 antitrypsin deficiency	Alpha-1-antitrypsin level	Normal
4	Alpha-1 antitrypsin deficiency	Alpha-1-antitrypsin level	Normal
5	Loeys-Dietz syndrome	Referral to a cardiologist	Seen by cardiologist, pending cardiologist letter
6	Loeys-Dietz syndrome	Referral to a cardiologist	Seen by cardiologist, pending cardiologist letter
7	Beckwith-Wiedemann syndrome	Discuss with patient	Not available
8	Alpha-1 antitrypsin deficiency	Alpha-1-antitrypsin level	Normal
9	DiGeorge syndrome—22q11 deletion syndrome	Referral to a clinical geneticist	Seen by clinical geneticist Microarray negative

## Discussion

While this pilot study was limited in scope, it demonstrated the potential of this approach in three critical areas:

1. It validated that a rare disease case-finding program could be designed and implemented following appropriate guidelines for data privacy and protection.
2. It demonstrated the feasibility of using phenotypes documented within primary care EHR as the basis for case finding algorithms.
3. It revealed that rare disease case finding is possible without significant disruption to the GP workflow or local specialist referral volumes.

There are two additional elements critical to the overall potential of EHR case finding that were not demonstrated in this pilot.

The first is the clinical validity of the algorithms in the form of statistically significant measures of accuracy such as positive predictive value, sensitivity and specificity. This pilot was not powered to demonstrate this and indeed given the low incidence of rare diseases, a very large sample size would be required to determine this for individual disease algorithms at conventional statistical significance. However, significant potential exists in future work to demonstrate the clinical validity of MendelScan’s multi-disease portfolio of algorithms as a broad rare disease tool. This is being supported by ongoing work to assess the analytical validity of individual disease algorithms in large research databases.

The second element is the scalability of this approach. To demonstrate this we will need to show the ability to:

- Gain direct access to dynamic EHR in an appropriate pseudonymised form.
- Demonstrate value to patients and efficiencies for GP. Further work to explore the acceptability and value of MendelScan to clinicians and patients is ongoing.
- Maintain the quality of returned reports with reduced reliance on the manual review process by Mendelian’s clinicians.

### Challenges and barriers for digital health deployment in rare diseases.

#### Data access considerations

A challenge to the adoption of digital health solutions often cited relates to information governance concerns such as data privacy, IT systems security and data confidentiality [20], with these concerns underpinned by the technical ability to hold the data safely and the dynamic aspect of the legal framework that governs this process.

In the current environment, it remains difficult to share and transfer patient health information between healthcare professionals from different organisations. This impairs the ability to create a seamless care environment that would improve the continuity of care across the different health care providers, a barrier to improving diagnostic delay.

A specific lesson from this pilot has been the need to establish a pathway to accelerate engagement with the final signatory stakeholders for information governance. In this pilot the initial contracting process and production of legal documentation took almost a year, as there were no standard documents for data transfer for commercial service provision. This is a reflection of the fact that commercial data access remains a relatively novel concept in the NHS, and highlights the need to establish clear criteria for commercial organisations to meet and a pathway for engagement to expedite this process.

#### Primary care EHR

To enable pseudo-anonymisation of the EHR data only structured/coded data was extracted. This structured data can be considered to be more accurate with numerical values such as weight, height, blood pressure and laboratory values readily comparable from one patient to the next. Diagnoses and clinical features are usually captured by physicians in UK primary care. Those that are encoded and therefore appearing in a structured data extract recorded, represent diagnoses that are recorded with a greater degree of confidence and therefore more reliable, with free text entries used by clinicians when they are less confident in the diagnosis. Consequently, one can consider the quality of this coded data as a strength. However, this means that the majority of information in the EHR was not examined by MendelScan. Free text diagnoses, clinical features and the content of correspondence, often rich in information, was not examined. For example, secondary care correspondence often rich in pertinent information may only generate two pieces of structured data: the presence of the letter and possibly a single diagnostic code. In addition, the motivators and context for coding in primary care needs to be considered, including the demonstration that certain tasks have been performed for reimbursement (e.g. Quality and Outcomes Framework) and to have pertinent information quickly to hand for future consultations.

#### Physician-related barriers for deployment

The implementation of any digital health tool into routine clinical practice faces challenges. However, MendelScan had some specific challenges related to working in the RD space and the timing of this pilot. Firstly, a lack of awareness that RD diagnosis is a primary care issue. The combined prevalence of RD, affecting 3.5–5.9% of the population [2], is not widely known and most GPs have seen relatively few RD patients.

Secondly, the sheer number of rare diseases can be daunting; this may lead the generalist to think that awareness of rare diseases is impossible [12]. However many features and presentations are shared, and empowering GPs to consider rare diseases as part of their diagnostic workup is an important component to impact rare disease diagnostic rates. MendelScan is one such solution, presenting to the clinician targeted information directly relevant to a specific patient, and equipping them with the information for their subsequent diagnostic work-up.

Thirdly, the restructuring of primary care organisations during the pilot led to further disruption with contracting, data-sharing agreements and responsibilities needing to change. Health care services are not static organisations and such changes need to be considered. Despite this, some of the restructuring changes such as the introduction of integrated care systems, an organisation challenged to improve public health across populations of 1–2 million, may be well placed for encouraging such rare disease case finding.

Fourthly, the COVID-19 pandemic significantly affected the implementation of this pilot, protracting its roll-out, and leading to practices dropping out due to additional clinical pressures. Despite this, we are optimistic that the wider use of routinely collected health data for research and quality improvement, and the rapid adoption of many digital health technologies that have occurred during the pandemic will help facilitate the use of technologies such as MendelScan in the future.

Finally, one cautionary lesson learned is that it is likely that the primary care federation data chosen for this pilot is better structured and the data stakeholders and system architecture more ready for the Mendelian approach than other primary care regions. More work needs to be done in prioritising which primary care healthcare systems have the capabilities to adopt MendelScan as a case-finding tool for RD.

## Conclusions

This pilot demonstrates that implementing a novel digital RD case-finding tool, MendelScan, in UK primary care is feasible, with minimal impact on workload or system resources. MendelScan identified credible cases, which subsequently were investigated in primary care or referred for further investigation. This study also highlighted challenges in implementing such a tool, including the restructuring of NHS organisations and shifting priorities due to outside pressures such as the COVID-19 pandemic.

Further research is ongoing, in the form of retrospective and prospective studies focusing on evaluating MendelScan’s analytical and clinical validity. Additional studies to evaluate the cost–benefit of early diagnosis, the impact of MendelScan on clinical practice and the acceptability and perception of this novel approach among specialists, GP and patients are in process or planned.

A digital health approach, such as MendelScan, could be an invaluable tool to address the rare disease diagnostic odyssey, flagging those potential zebras amongst the horses. In addition, through the use of routinely collected EHR data, it can be scaled quickly and adopted broadly helping to ensure equality of access to a correct and timely diagnosis.

## Data Availability

Data sharing is not possible for this article as no datasets were generated or analysed during the current study.

## Appendix

1. Rare diseases algorithms

**Table:**

Addison’s disease
Alkaptonuria
Alpers-Huttenlocher syndrome
Alpha-1 antitrypsin deficiency
Alpha-mannosidosis
Alport syndrome
Alström syndrome
Amaurosis congenita of Leber
Apert syndrome
Argininosuccinate lyase deficiency
Atypical osteogenesis imperfecta
Bardet-Biedl syndrome
Barth syndrome
Beckwith-Wiedemann syndrome
Behcet’s disease
Beta kethiolase deficiency
Carcinoid syndrome
Ceroid lipofuscinosis, neuronal
Charcot-Marie-Tooth disease
Chronic progressive external ophthalmoplegia (CPEO)
Citrullinemia I and II
Classic homocystinuria
Coffin-Lowry syndrome
Common variable immunodeficiency (CVID)
Cystinosis
Deficiency of long chain 3-hydroxyacyl-CoA dehydrogenase
Dermatomyositis
DiGeorge syndrome—22q11 deletion syndrome
Duane syndrome
EDS arthochalasia
EDS myopathic
Ehlers Danlos syndrome
Fabry disease
Fibrodysplasia ossificans progressiva
Fragile × syndrome
Gaucher disease
Glycogen storage disease type 2 (Pompe)
Glycogen storage disease type 5 (Mcardle)
Good syndrome
Hartnup disease
Hereditary angioedema
Insulinoma
Isovaleric acidemia
Li-Fraumeni syndrome
Loeys-Dietz syndrome
Lowe Syndrome
Marfan syndrome
Maternally inherited diabetes and deafness
MELAS syndrome
Metachromatic leukodystrophy
Mitochondrial diseases
Mucopolysaccharidosis
Myoclonic epilepsy with ragged red fibers (MERRF)
Neurofibromatosis type 1
Neurofibromatosis type 2
Neuropathy, ataxia, and retinitis pigmentosa (NARP)
Niemann-Pick B disease
Niemann-Pick C disease
Porphyria, acute intermittent
Porphyria, erythropoietic
Prader-Willi syndrome
Primary biliary cirrhosis
Primary Hyperoxaluria
Propionic acidemia
PTEN hamartoma tumour syndrome
Rendu-Osler-Weber disease (hereditary hemorrhagic telangiectasia)
Scleroderma
Sensory ataxic neuropathy-dysarthria-ophthalmoparesis syndrome (SANDO)
Stickler syndrome
Sucrase-isomaltase deficiency
Transthyretin (TTR) amyloidosis
Tuberous sclerosis
Turner syndrome
Von Hippel-Lindau disease
William syndrome
Wolfram syndrome

(2) Feedback questionnaire

**Table:**

#	Feedback questions (patient report feedback)
1	Please indicate the first 5 characters of the patient's encrypted NHS number
2	Please indicate which of the options below best describe this report. Tick box multiple answer questions Reasonable possible diagnosis (advance for further evaluation) Diagnosis has already been excluded Patient has clear alternative diagnosis Other (patient left study GP locality/unable to accurate identify patient)
3	Please indicate what action was or is planned to be taken by the GP. Tick box multiple answer questions Reviewed patient's medical history Added a note to review the patient on their next visit Recalled the patient for additional history taking and/or physical examination (include details in the comments below) Requested an additional test (include details in the comments below) Referred the patient to a specialist/secondary care (include details in the comments below) Discussed the patient with an expert (include the expert's name in the comments below) No action taken (include details in the comments below) Other
4	In order for us to minimise the time it takes to review cases. Please indicate how long it has taken you to review this case and reach your decision 0–5 min 5–15 min 15–30 min > 30 min
5	In order to ensure we constantly improve, please add any comments or remarks about how you have found the process. (e.g. Was it straightforward? Did you find it interesting? What could be improved?) *(free text)*
